# An improved YOLOv7 model based on Swin Transformer and Trident Pyramid Networks for accurate tomato detection

**DOI:** 10.3389/fpls.2024.1452821

**Published:** 2024-09-26

**Authors:** Guoxu Liu, Yonghui Zhang, Jun Liu, Deyong Liu, Chunlei Chen, Yujie Li, Xiujie Zhang, Philippe Lyonel Touko Mbouembe

**Affiliations:** ^1^ School of Computer Engineering, Weifang University, Weifang, China; ^2^ Shandong Provincial University Laboratory for Protected Horticulture, Weifang University of Science and Technology, Weifang, China; ^3^ School of Computer Science, Weifang University of Science and Technology, Weifang, China; ^4^ Department of Electronics Engineering, Pusan National University, Busan, Republic of Korea

**Keywords:** tomato detection, YOLOv7, Swin Transformer, Trident Pyramid Network, Focaler-IoU

## Abstract

Accurate fruit detection is crucial for automated fruit picking. However, real-world scenarios, influenced by complex environmental factors such as illumination variations, occlusion, and overlap, pose significant challenges to accurate fruit detection. These challenges subsequently impact the commercialization of fruit harvesting robots. A tomato detection model named YOLO-SwinTF, based on YOLOv7, is proposed to address these challenges. Integrating Swin Transformer (ST) blocks into the backbone network enables the model to capture global information by modeling long-range visual dependencies. Trident Pyramid Networks (TPN) are introduced to overcome the limitations of PANet’s focus on communication-based processing. TPN incorporates multiple self-processing (SP) modules within existing top-down and bottom-up architectures, allowing feature maps to generate new findings for communication. In addition, Focaler-IoU is introduced to reconstruct the original intersection-over-union (IoU) loss to allow the loss function to adjust its focus based on the distribution of difficult and easy samples. The proposed model is evaluated on a tomato dataset, and the experimental results demonstrated that the proposed model’s detection recall, precision, F_1_ score, and AP reach 96.27%, 96.17%, 96.22%, and 98.67%, respectively. These represent improvements of 1.64%, 0.92%, 1.28%, and 0.88% compared to the original YOLOv7 model. When compared to other state-of-the-art detection methods, this approach achieves superior performance in terms of accuracy while maintaining comparable detection speed. In addition, the proposed model exhibits strong robustness under various lighting and occlusion conditions, demonstrating its significant potential in tomato detection.

## 1 Introduction

Fruit harvesting is a critical step in the agricultural production process. However, traditional manual methods are costly, time-consuming, and inefficient, complicating meeting large-scale cultivation demands. Due to the advancement of smart agriculture, the transition from manual labor to automated fruit harvesting has become an inevitable trend. For fruit harvesting robots, accurate fruit identification and localization are essential for efficient harvesting. Therefore, it is very important to develop robust and accurate fruit detection algorithms for the robotic vision systems.

Over the past few decades, numerous researchers have explored various fruit detection methods. These approaches are generally categorized into threshold discrimination and machine learning-based methods. Initially, the fruit targets in images are segmented by setting thresholds based on simple features such as color (Wei et al., 2014), shape (Kelman and Linker, 2014), texture (Rakun et al., 2011), or a combination of these features (Payne et al., 2014), to complete the detection process. Although these methods yield reasonable results, the sensitivity of the thresholds to environmental variations limits their generalization capabilities. The introduction of machine learning has mitigated these limitations. Traditional techniques, which integrate handcrafted features such as Histogram of Oriented Gradients and Haar features with machine learning models like Support Vector Machine (SVM) (Liu et al., 2019) and AdaBoost (Zhao et al., 2016), have been employed to locate and recognize fruits. Following the success of deep learning in computer vision (Krizhevsky et al., 2012), it has been applied to smart agriculture (Sa et al., 2016; Fuentes et al., 2017). Deep learning models are adept at directly extracting features from data and facilitating end-to-end training, significantly enhancing the models’ detection performance and efficiency.

Despite the significant advancements in deep learning-based fruit detection methods, several shortcomings persist. These models are typically trained on data from controlled conditions, resulting in reduced robustness against unconstrained factors in real-world environments, such as illumination variations and occlusion or overlap occurrences. In addition, the traditional IoU-based regression loss function utilized in the YOLO model cannot accurately predict the position of fruit targets. Due to the limitations inherent in traditional regression methods, which neglect the distribution of objects across different scales, they can fail to accurately identify the location of fruit targets, particularly in challenging scenarios.

In order to address these challenges, this study introduces a novel YOLO-SwinTF model, designed to enhance the accuracy of tomato detection in complex environments while maintaining high detection efficiency. Based on the YOLOv7 architecture, the model’s backbone, neck, and loss function are refined to improve feature extraction and target-focusing capabilities. Specifically, Swin Transformer blocks are incorporated into the backbone to aid the model in capturing long-range visual dependencies while maintaining computational efficiency, thereby enhancing the semantic information of the features. Then, the original PANet is replaced with the TPN architecture by embedding multiple SP modules between the traditional top-down and bottom-up architectures. This modification allows the feature mapping to generate new information for propagation. In addition, a Focaler-IoU loss is constructed using a linear interval mapping method to adjust its focus based on sample difficulty, improving the model’s detection performance.

The main contributions to this study are as follows:

1. A novel network architecture, YOLO-SwinTF, is proposed, which incorporates the Swin Transformer attention mechanism and Trident Pyramid Network architectures to enhance feature extraction capabilities.
2. The Focaler-IoU loss is introduced to accurately identify tomato locations. This method enhances the detection performance of the model by dynamically adjusting the focus of the loss among samples of varying difficulty.
3. Extensive experiments on tomato datasets demonstrate that the proposed YOLO-SwinTF model achieves excellent performance compared to the current state-of-the-art methods for tomato detection.

The remainder of this paper is organized as follows: Section 2 reviews the literature on fruit detection methods, which include threshold-based discriminant analysis, machine learning, and deep learning approaches. Section 3 introduces the proposed tomato detection model. The experimental results obtained through the proposed method are presented and discussed in Section 4. Finally, Section 5 concludes the study.

## 2 Related work

### 2.1 Threshold-based discriminant methods

In the early days, researchers employed simple features such as color, shape, and texture to detect fruits. Kurtulmus et al. (2011) developed a method for detecting and counting green citrus fruits in natural environments using color images. They introduced a novel “eigenfruit” approach that incorporated color, circularity, and Gabor texture analysis to identify the fruits. Then, a shifting sub-window technique was applied at three different scales to scan the image and localize the fruits. In their study, 73% of green fruits were correctly identified. Ji et al. (2012) established an automatic vision recognition system to guide apple harvesting robots. Images of the apples were captured using a color charge-coupled device camera. An industrial computer processed and recognized the apples. A vector median filter removed noise from the color images of the apples, and an image segmentation algorithm based on region and color features was applied. The study reported an accuracy of 89% with an average detection time of 352 ms. Chaivivatrakul and Dailey (2014) developed a texture-based fruit detection approach. This method utilizes interest-point feature extraction and descriptor computation. A low-cost web camera suitable for mechanized systems evaluated 24 combinations of interest-point features and descriptors for pineapples and bitter melons. The method achieved an accuracy of 85% for the single-image detection of pineapples and 100% for bitter melons. Jana and Parekh (2017) proposed a shape-based fruit recognition approach, which included a pre-processing step that normalizes fruit images to account for translation, rotation, and scaling differences. This method then employed features unaffected by variations in distance, growth phase, and surface appearance of the fruits for detection. The proposed method was applied to a dataset of 210 images covering seven different fruit classes, achieving an overall recognition accuracy between 88% and 95%.

Although threshold-based discriminant methods have demonstrated reasonable effectiveness in detecting fruits, their performance significantly depends on the appropriateness of the selected thresholds. This dependence can result in limited model generalization and diminish detection robustness.

### 2.2 Traditional machine learning-based methods

Due to the development of machine learning, many researchers have attempted to apply it to fruit detection. Methodologies include Adaboost (Payne et al., 2014), Random Forests (Samajpati and Degadwala, 2016), and SVM (Behera et al., 2020). Using machine vision and SVM, Peng et al. (2018) conducted a study on detecting different classes of fruit, such as apples, bananas, citruses, carambolas, pears, and pitaya. The process involved using a Gaussian filter and histogram equalization for image processing, followed by segmentation with the Otsu method. To extract features, researchers employed shape-invariant moments and synthesized the color and shape of fruits. An SVM was then used to classify and detect the fruits, achieving detection rates of 95% for apples, 80% for bananas, 97.5% for citrus fruits, 86.7% for carambola, 92.5% for pears, and 96.7% for pitaya. Jiao et al. (2020) proposed a detection and localization method for overlapping apples, which began with the transformation and segmentation of apple images using the Lab color space and K-means algorithm. Morphological processes such as erosion and dilation were applied to delineate the apple edges. In addition, a fast algorithm calculated the minimum distance from each interior point to the apple outline, determining the radii by identifying the shortest distance from the center to the edge. Zhu et al. (2021) developed a carrot detection method by extracting deep features from a three-layer fully connected layer of network models and integrating these with an SVM. Their most effective model combined ResNet101 with an SVM, achieving an accuracy of 98.17%. Yu et al. (2021) proposed a method for identifying ripe litchi using an RGB-D camera in natural environments. Their approach utilized both color and depth images for litchi detection. Initially, depth image segmentation was employed to eliminate redundant image information outside the effective range of the manipulator. A random forest binary classification model was then trained using color and texture features to detect litchi fruits, achieving detection accuracies of 89.92% for green litchis and 94.50% for red litchis.

Although machine learning has significantly advanced fruit detection, these methods predominantly rely on handcrafted features and possess inherent limitations. Their capacity to abstract features is restricted, confining them to simple scenarios and limiting their generalization capabilities. In addition, the models lack end-to-end learning, which diminishes learning efficiency.

### 2.3 Deep learning-based methods

In recent years, deep learning-based approaches have emerged as powerful alternatives. In particular, convolutional neural networks (CNN) have shown remarkable success in learning discriminative features directly from raw image data without needing handcrafted features. CNN-based architectures such as Faster R-CNN (Ren et al., 2015), YOLO (Redmon et al., 2016; Redmon and Farhadi, 2017, 2018; Bochkovskiy et al., 2020; Wang et al., 2023), and SSD (Liu et al., 2016) have been widely used for fruit detection. Bargoti and Underwood (2017) proposed a deep model for detecting fruits in orchards, based on Faster R-CNN (Ren et al., 2015), to detect mangoes, almonds, and apples. This method achieved an F_1_ score of 90% for mangoes and apples. Ganesh et al. (2019) utilized Mask R-CNN (He et al., 2017) to detect individual fruits and obtain pixel-wise masks for each detected fruit in an image, achieving an overall F_1_ score of approximately 89%. Despite the advancements in two-stage methods that use separate networks to predict bounding boxes and class probabilities from an input image, these are not well suited for real-time applications. Recently, YOLO algorithms have been proposed to address this issue using a single CNN to predict and classify objects. Hernández et al. (2023) developed a tomato detection and classification method based on YOLOv3-tiny (Redmon and Farhadi, 2018), achieving an F_1_ score of 90% for detecting ripe tomatoes. Guo et al. (2023) employed YOLOv7 for the real-time detection of ripe tomatoes, using an improved RepLKNet (Ding et al., 2022) to enhance the receptive field. In addition, the head structure of YOLOv7 was redesigned to address the issue of low FLOPS, and FasterNet (Chen et al., 2023) was used to optimize the structure between the Concat and CBS in the head. ODConv (Li et al., 2022) was added to improve the feature extraction for small tomatoes, achieving an mAP (0.5:0.95) of 56.8% with a detection time of 0.0127 s. Zeng et al. (2023) proposed a lightweight modified YOLOv5 for real-time localization and ripeness detection of tomatoes, achieving an mAP of 96.9% with a detection speed of 42.5 ms. Mbouembe et al. (2023) developed an efficient tomato detection method based on YOLOv4, incorporating an improved BottleneckCSP, a modified CSP-SPP, and CARAFE into the YOLOv4 architecture to enhance the feature expression capabilities of the model. This method achieved an mAP of 98.5%. Wang et al. (2024c) developed a grape detection algorithm based on YOLOv5s, introducing a dual-channel feature extraction attention mechanism (Li et al., 2017) and a dynamic snake convolution (Qi et al., 2023) in the backbone network to improve feature extraction. The mAP (0.5:0.95) was 69.3%. Gao et al. (2024) established an improved binocular calyx localization method based on YOLOv5x to detect kiwifruit, achieving an mAP of 93.5% with a detection speed of 105 ms per image.

Despite advances in deep learning-based fruit detection, several challenges remain. Variability in fruit appearance due to uneven illumination, overlap, and occlusion poses a challenge for accurate detection. In addition, the presence of similar-looking objects and background clutter further complicates this task.

## 3 Materials and methods

### 3.1 Image acquisition

**Figure 1:**
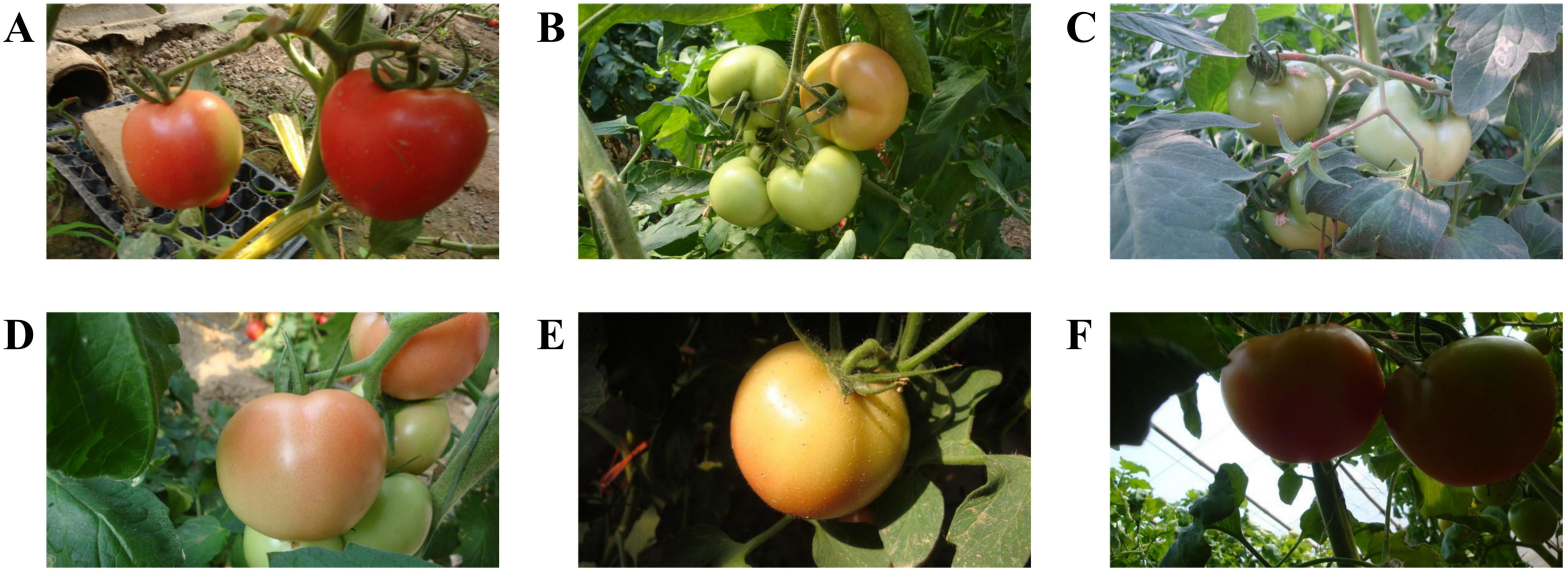
Tomato samples with different growing circumstances: **(A)** separated tomatoes, **(B)** a cluster of tomatoes, **(C)** occlusion case, **(D)** overlap case, **(E)** sunlight case, and **(F)** shade case.

The tomato dataset for this study was collected at the Shouguang Vegetable High-Tech Demonstration Park in Shandong Province, China between 2017 and 2019. The acquisition equipment utilized was a Sony digital camera (Sony DSC-W170, Tokio, Japan) with a resolution of 3648 × 2056 pixels. This study collected 966 tomato images under various environmental conditions, including sunlight, shade, overlap, and occlusion. Considering that the dataset is not large, additional splitting could lead to a smaller training set, which is prone to overfitting (Ashtiani et al., 2021). Therefore, we divided the data into training and test sets at a ratio of 3:1, following (Liu et al., 2022; Jia et al., 2023). The training dataset comprised 725 images featuring 2553 tomatoes, whereas the test set included 241 images with 912 tomatoes. **Figure 1** displays a selection of example images captured under various environmental conditions.

### 3.2 Image augmentation

**Figure 2:**
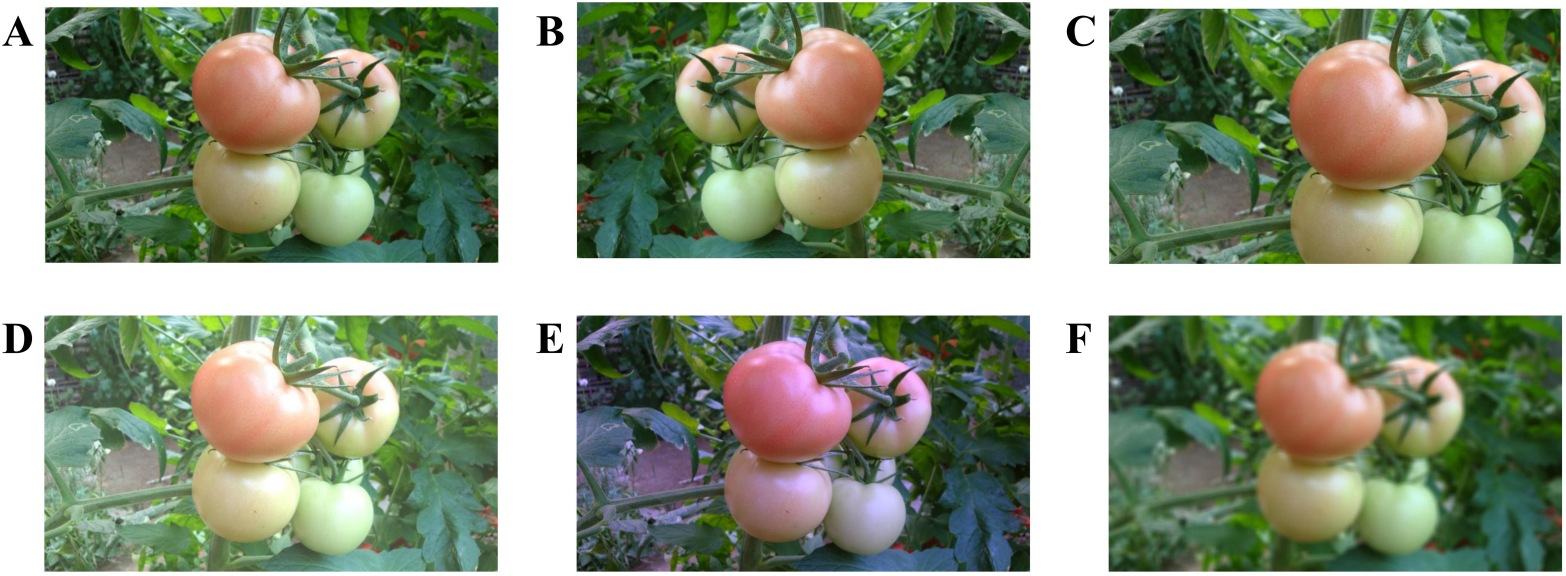
Data augmentation of tomato images: **(A)** original image, **(B)** horizontal flip, **(C)** scaling and cropping, **(D)** brightness transformation, **(E)** color balancing, and **(F)** image blurring.

The study applied data augmentation techniques to the collected images to enhance the generalization capability of the trained model and prevent overfitting. This resulted in a final set of 4350 enhanced images, achieved through horizontal flipping, scaling and cropping, brightness transformation, color balancing and blurring, as shown in **Figure 2**. For brightness transformation, a random factor ranging from 0.6 to 1.4 was employed to modulate pixel intensity, simulating the effects of diverse weather conditions on image brightness. Scaling and cropping were performed according to the methods described by Liu et al. (2020). During this phase, images without labels were discarded. The Gray World algorithm (Lam, 2005) was employed for color balancing to mitigate the impact of lighting on color rendering. Then, random blurring was applied to the augmented images to mimic the indistinct visuals that can result from camera motion. **Table 1** lists the total number of resulting images after data augmentation.

**Table 1:** The number of training images after data augmentation.

	Original	Honrizontal flip	Scaling and cropping	Brightness transformation	Color balancing	Blurring	Total
No. of images	725	725	725	725	725	725	4350

### 3.3 YOLOv7 model

YOLOv7 (Wang et al., 2023) is an anchor-based detection method among the widely used YOLO algorithms. Like other iterations in the YOLO series, this version comprises three components: a backbone network for feature extraction; a neck network that fuses and refines the extracted features, yielding large, medium, and small feature sets; and a head network that utilizes these features from the neck to generate prediction outputs.

YOLOv7 developed a new backbone network called EfficientRep, which is a redesigned and improved version of the EfficientNet architecture (Tan and Le, 2019). This new backbone network includes different modules: E-ELAN, MPConv, and SPPCSPC. The E-ELAN module is an extended version of the ELAN (Wang et al., 2022). The original ELAN was designed to address the problem of convergence in deep models, which can gradually deteriorate as the models scale. E-ELAN maintains the same gradient flow as ELAN, but increases cardinality through group convolution. The MPConv module strikes a balance between increasing representational capacity and maintaining computational efficiency. The SPPCSPC module is a combination of the SPP module (He et al., 2015) and the CSP module (Wang et al., 2020). The SPP module captures features at different spatial resolutions, which is beneficial for detecting objects of various sizes. The CSP module then facilitates the flow of information between different stages and concatenates the output of the SPP module with the previous stage’s feature maps, creating a richer and more diverse feature representation.

The neck network combines relevant feature maps from the backbone network using the PANet architecture (Liu et al., 2018) for feature fusion. In addition, YOLOv7 uses the RepConv technique (Ding et al., 2021) to address the challenges of detecting objects at various scales by enhancing the representability of feature maps. This technique also improves the inference results, although it increases the training time by introducing gradient diversity and allowing for more complex feature representations.

The head network uses anchor boxes to predict the objects’ position, size, and class in the input image. Subsequently, a post-processing technique known as Non-Maximum Suppression (NMS) is employed to refine the predicted object boxes by eliminating redundant detections, enhancing the accuracy of YOLOv7.

### 3.4 The proposed YOLO-SwinTF

**Figure 3:**
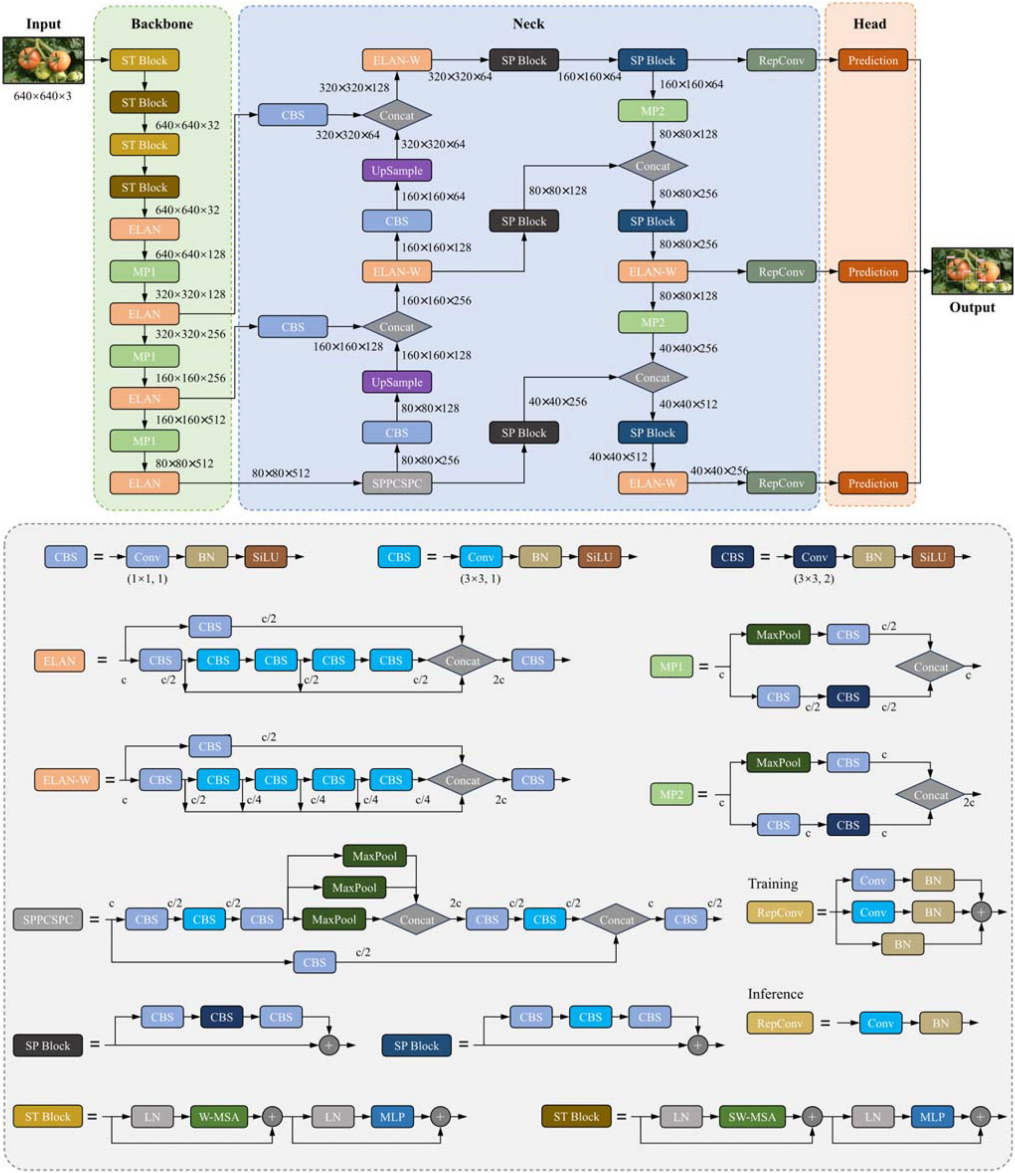
The architecture of the proposed YOLO-SwinTF.

This study introduces the YOLO-SwinTF model, an advancement based on YOLOv7, to enhance the accuracy and robustness of tomato detection in complex environments. **Figure 3** illustrates the architecture of the proposed YOLO-SwinTF model. It integrates three innovative modules to enhance the feature expression capability, improving the detection accuracy. Initially, ST blocks were incorporated into the backbone network, enabling the model to capture long-range dependencies efficiently. Subsequently, the TPN architecture replaced the original PANet in the neck network. This replacement was achieved by embedding multiple SP modules within the existing top-down and bottom-up architectures, facilitating the generation and effective propagation of new information within the feature maps. Finally, a Focaler-IoU loss was constructed using a linear interval mapping method. This method dynamically adjusts its focus based on the difficulty of the samples, significantly enhancing the detection capabilities of the model. Further details are provided in Sections 3.4.1 – 3.4.4.

#### 3.4.1 Swin Transformer block

**Figure 4:**
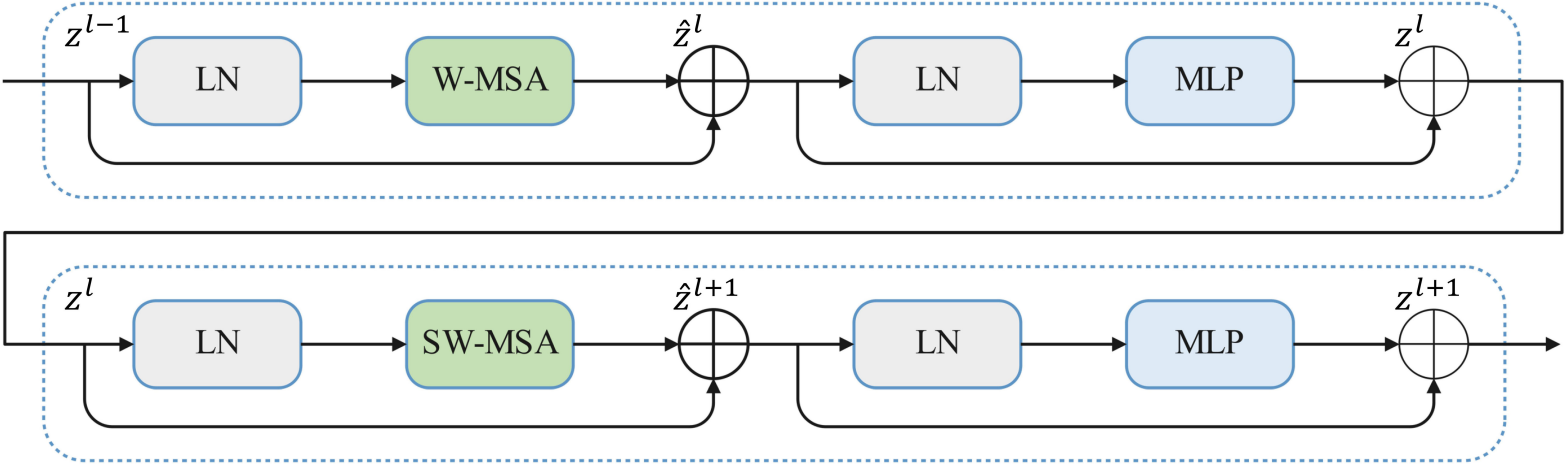
The Swin Transformer blocks.

Although CNN networks can effectively extract local features, they are limited in capturing global features, impacting the final detection performance. In order to address this limitation, the current study introduces the attention mechanism of the Swin Transformer (Liu et al., 2021) to enhance the model’s long-range dependencies. Unlike traditional Transformer structures, the Swin Transformer employs a hierarchical attention mechanism. In this structure, a sliding window performs attention computations separately at different layers, diverging from the standard multi-head self-attention (MSA) module. This approach not only facilitates the extraction of global information through long-distance modeling but also reduces the computational complexity of the original attention method. **Figure 4** indicates that a Swin Transformer module primarily comprises a LayerNorm (LN) layer, a window-based multi-head self-attention (W-MSA) module, a shifted window-based multi-head self-attention (SW-MSA) module, a two-layer multi-layer perceptron (MLP) with a GELU non-linear activation function between layers, and a residual connection.

**Figure 4** shows that two consecutive Swin Transformer blocks are computed using Equations 1-4 (Liu et al., 2021):


(1) $${\hat{z}}^{l}=W-MSA\left(LN\left(z^{l-1}\right)\right)+z^{l-1}$$

(2) $$z^{l}=MLP\left(LN\left({\hat{z}}^{l}\right)\right)+{\hat{z}}^{l}$$

(3) $${\hat{z}}^{l+1}=SW-MSA\left(LN\left(z^{l}\right)\right)+z^{l}$$

(4) $$z^{l+1}=MLP\left(LN\left({\hat{z}}^{l+1}\right)\right)+{\hat{z}}^{l+1}$$

where ${\hat{z}}^{l}$ denotes the output of the (S)W-MSA module and $z^{l}$ denotes the output of the MLP module of the *l*th block.

In order to enable the model to capture global information, the first four CBS modules in YOLOv7 were replaced with four successive ST blocks, thus expanding the network’s receptive field and enriching contextual information, as depicted in **Figure 3**.

#### 3.4.2 Trident Pyramid Network architecture

As discussed by Picron and Tuytelaars (2022), existing feature pyramid networks (FPN, PANet, and BiFPN) primarily focus on communication-based processing, enhancing feature fusion through top-down and bottom-up operations. These networks can become saturated with communication when multiple communication-based operations are performed consecutively, reducing efficiency. Accordingly, this study introduces the TPN architecture to replace PANet in YOLOv7, which achieves a better balance between communication-based processing and self-processing by alternating top-down and bottom-up operations and parallel self-processing mechanisms.

**Figure 5:**
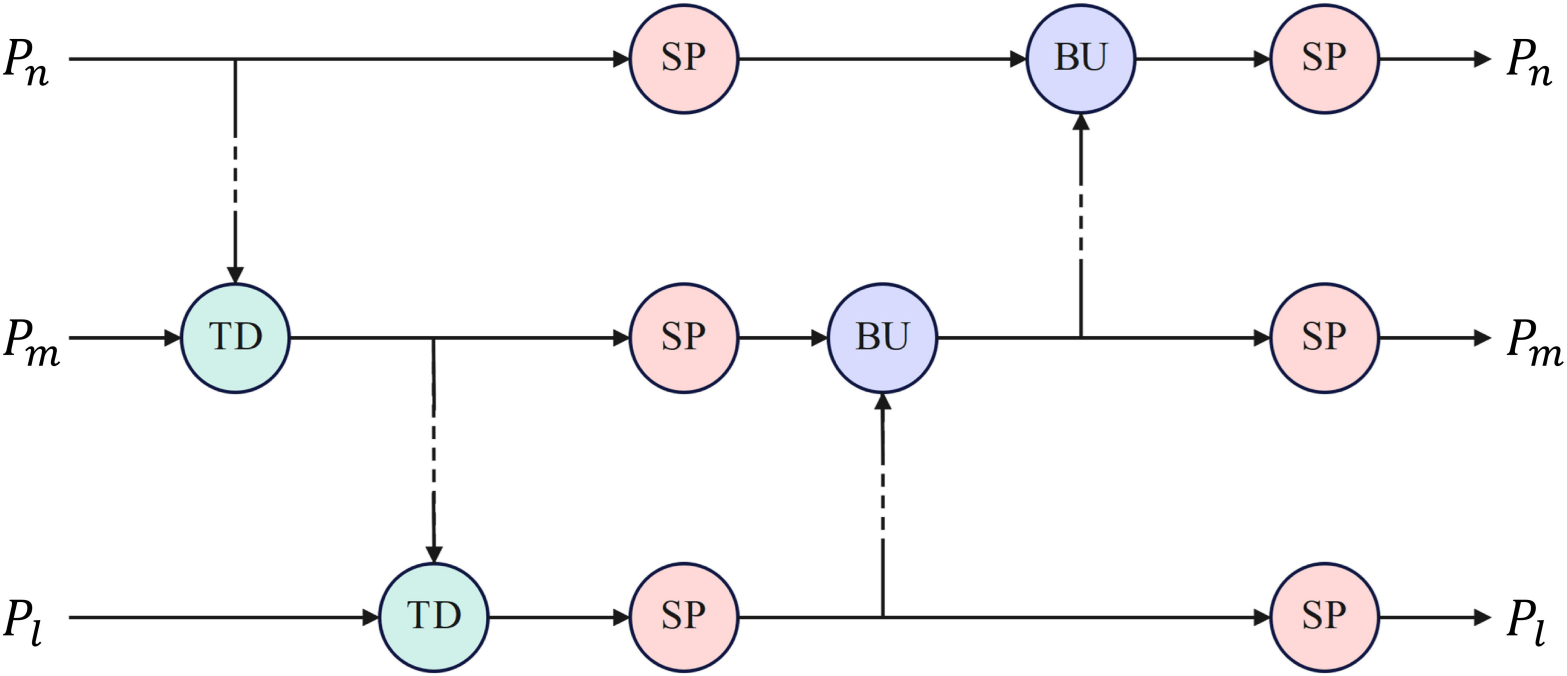
The TPN architecture. TD, BU and SP denotes top down, bottom up and self-processing modules, respectively.

**Figure 6:**
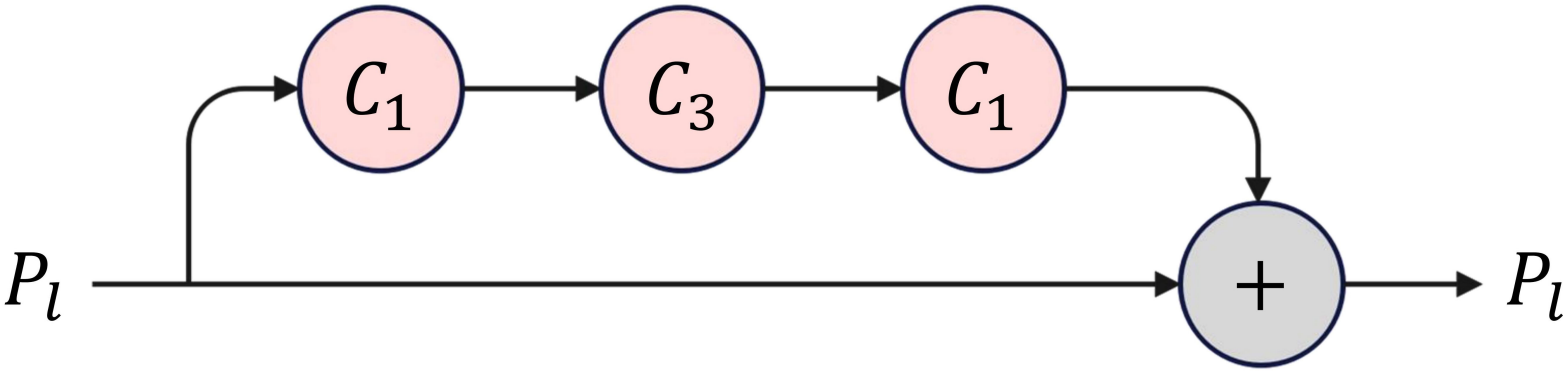
The architecture of a base self-processing layer. *C*_1_ and *C*_3_ denote convolution operations with kernel sizes of 1 and 3, respectively.

Specifically, the TPN architecture consists of traditional top-down and bottom-up operations and parallel SP modules, as illustrated in **Figure 5**. An SP module consists of several consecutive base self-processing layers, each designated as a bottleneck layer, as depicted in **Figure 6**.

Multiple SP modules were explicitly embedded between the original top-down and bottom-up architectures. As shown in **Figure 3**, the SP module was added after the SPPCSPC and ELAN-W modules in the bottom-up architecture. In addition, the SP module processed the features again after being merged into the top-down architecture. In this manner, communication-based processing is alternated with self-processing, enabling feature mapping to generate new information for delivery. The TPN architecture controls the amount of self-processing through the hyperparameter, the number of layers in the SP module, *N*, which is set to 2 in this study.

#### 3.4.3 Focaler-IoU-based regression loss

The accuracy of bounding box localization is critical to target detection performance. However, existing studies often overlook the impact of the distribution of difficult samples (small targets that are difficult to accurately localize) and easy samples (targets that are easy to detect) on bounding box regression. This oversight can result in suboptimal performance and a lack of robustness in challenging scenarios. To address this issue, this study introduces Focaler-IoU (Zhang and Zhang, 2024) to enhance detector performance in the tomato detection task by effectively focusing on different regression samples.

Specifically, the Focaler-IoU reconstructs the original IoU loss through a linear interval mapping method that allows the loss function to adjust its focus according to the distribution of difficult and easy samples. The reconstructed Focaler-IoU $IoU^{focaler}$ is expressed as follows (Zhang and Zhang, 2024):


(5) $$IoU^{focaler}=\{\begin{array}{ll} 0, & IoU<d\\ \frac{IoU-d}{u-d}, & d\leq IoU\leq u\\ 1, & IoU>u \end{array}$$

where IoU is the original IoU value, and *d* and *u* are both in the range of [0,1]. Adjusting the values of *d* and *u* can guide $IoU^{focaler}$ to focus on different regression samples. In this study, *d* and *u* were set to 0.1 and 0.9, respectively. Accordingly, the Focaler-IoU loss $L_{Focaler-IoU}$ is defined below:


(6) $$L_{Focaler-IoU}=\;1\;–IoU^{focaler}$$

Referring to Zhang and Zhang (2024), the Focaler-IoU loss is applied to the original CIoU-based bounding box regression loss used in YOLOv7, resulting in a novel regression loss as follows:


(7) $$L_{reɡ}=L_{CIoU}+IoU-IoU^{focaler}$$

Where $L_{CIoU}$ is expressed as follows (Zheng et al., 2020):


(8) $$L_{CIoU}=1-IoU+\frac{d^{2}\left(b,b_{ɡt}\right)}{c^{2}}+\beta v$$

where d(·) denotes Euclidean distance. b and $b_{ɡt}$ denote the central points of the predicted and ground truth bounding boxes, respectively. β represents a positive trade-off parameter and v quantifies the consistency of the aspect ratio, as detailed below.


(9) $$v=\frac{4}{\pi^{2}}{\left(\text{arctan }\frac{w_{ɡt}}{h_{ɡt}}-\text{arctan }\frac{w}{h}\right)}^{2}$$

(10) $$\beta =\frac{v}{\left(1-IoU\right)+v}$$

Combining Equations 7 and 8, we obtain the final regression loss as follows:


(11) $$L_{reɡ}=1-IoU^{focaler}+\frac{d^{2}\left(b,b_{ɡt}\right)}{c^{2}}+\beta v$$

This approach enables the loss function to dynamically adjust its focus between easy and difficult samples, enhancing the performance of the model in the detection task. Simultaneously, the adjustment of the loss function allows the model to concentrate more on positive samples that are difficult to classify and less on negative samples that are easy to classify. This adjustment effectively improves the model’s response to the imbalance between difficult and easy samples.

#### 3.4.4 Loss function

As in YOLOv7 (Wang et al., 2023), the loss function of the proposed model consists of three parts, i.e., the regression loss $L_{reɡ}$ , confidence loss $L_{conf}$ , and classification loss $L_{cls}$ , and is expressed as follows:


(12) $$L_{total}={\text{λ}}_{reɡ}L_{reɡ}+{\text{λ}}_{conf}L_{conf}+{\text{λ}}_{cls}L_{cls}$$

where ${\text{λ}}_{reɡ}$ , ${\text{λ}}_{conf}$ and ${\text{λ}}_{cls}$ were set to 5, 1, and 1, respectively, to balance the different losses. $L_{reɡ}$ , $L_{conf}$ , and $L_{cls}$ are expressed in Equations 11, 13 and 14, respectively.


(13) $$\begin{array}{c} L_{conf}=\sum_{i=1}^{s\times s}\sum_{j=1}^{NB}\lambda_{i,j}[-C_{i}\text{log }{\tilde{C}}_{i}]\\ \;\;\;\sum_{i=1}^{s\times s}\sum_{j=1}^{NB}(1-\lambda_{i,j})[-(1-C_{i})\text{log }(1-{\tilde{C}}_{i})] \end{array}$$

(14) $$L_{cls}=\sum_{i=1}^{s\times s}\sum_{j=1}^{NB}\lambda_{i,j}\sum_{a\in \text{classes }}\left[p_{i}\left(a\right)\text{log }{\tilde{p}}_{i}\left(a\right)+\left(1-p_{i}\left(a\right)\right)\text{log }\left(1-{\tilde{p}}_{i}\left(a\right)\right)\right]$$

where s×s denotes the grid cell size, and *NB* is the number of bounding boxes. ${\tilde{C}}_{i}$ and $C_{i}$ represent the confidence of the predicted box and the confidence threshold, respectively. $\lambda_{i,j}$ equals 1 if the *j*th bounding box falls in the *i*th grid cell and 0 otherwise. ${\tilde{p}}_{i}$ and $p_{i}$ denote the predicted and ground truth class probabilities, respectively.

## 4 Experimental results and discussion

### 4.1 Experimental environment

Our experiments were conducted on a server with a 43GB Intel(R) Xeon(R) Platinum 8255C CPU operating at 2.50GHz and an NVIDIA GeForce RTX 3090 GPU. The server runs Ubuntu 18.04 as its underlying operating system. The proposed model was implemented using the PyTorch framework.

The model was trained with an input resolution of 640 × 640 pixels and a batch size of 32. The SGD optimizer was employed for training with a momentum of 0.937 and a weight decay of 0.0005. A cosine annealing schedule was applied to control changes in learning rates, starting with an initial learning rate of 0.001. The training was carried out over 160 epochs. The hyperparameters used in this study are listed in **Table 2**.

**Table 2:** The hyperparameter settings of YOLO-SwinTF.

Hyperparameter	Value
Initial learning rate	0.001
Weight decay	0.0005
Momentum	0.937
Batch size	32
Epochs	160

### 4.2 Evaluation metrics

For a thorough evaluation of the performance of the proposed method, the recall (R), precision (P), and F_1_ score (Sa et al., 2016) were adopted as evaluation metrics. These metrics are defined as follows.


(15) $$P=\frac{TP}{TP+FP}$$

(16) $$R=\frac{TP}{TP+FN}$$

(17) $$F_{1}=\frac{2\times P\times R}{P+R}$$

where TP, FP, and FN denote true positive (correct detection), false positive (false detection), and false negative (missing detection), respectively.

In addition, this study employed Average Precision (AP) (Everingham et al., 2010) to assess the overall performance of the detection system. AP is defined as follows:


(18) $$AP=\sum_{n}\left(r_{n+1}-r_{n}\right)p_{interp}\left(r_{n+1}\right)$$

(19) $$p_{interp}\left(r_{n+1}\right)=\underset{\tilde{r}:\tilde{r}\geq r_{n+1}}{\max}p\left(\tilde{r}\right)$$

where $p\left(\tilde{r}\right)$ is the measured precision at a recall level of $\tilde{r}$ .

### 4.3 Ablation study

**Figure 7:**
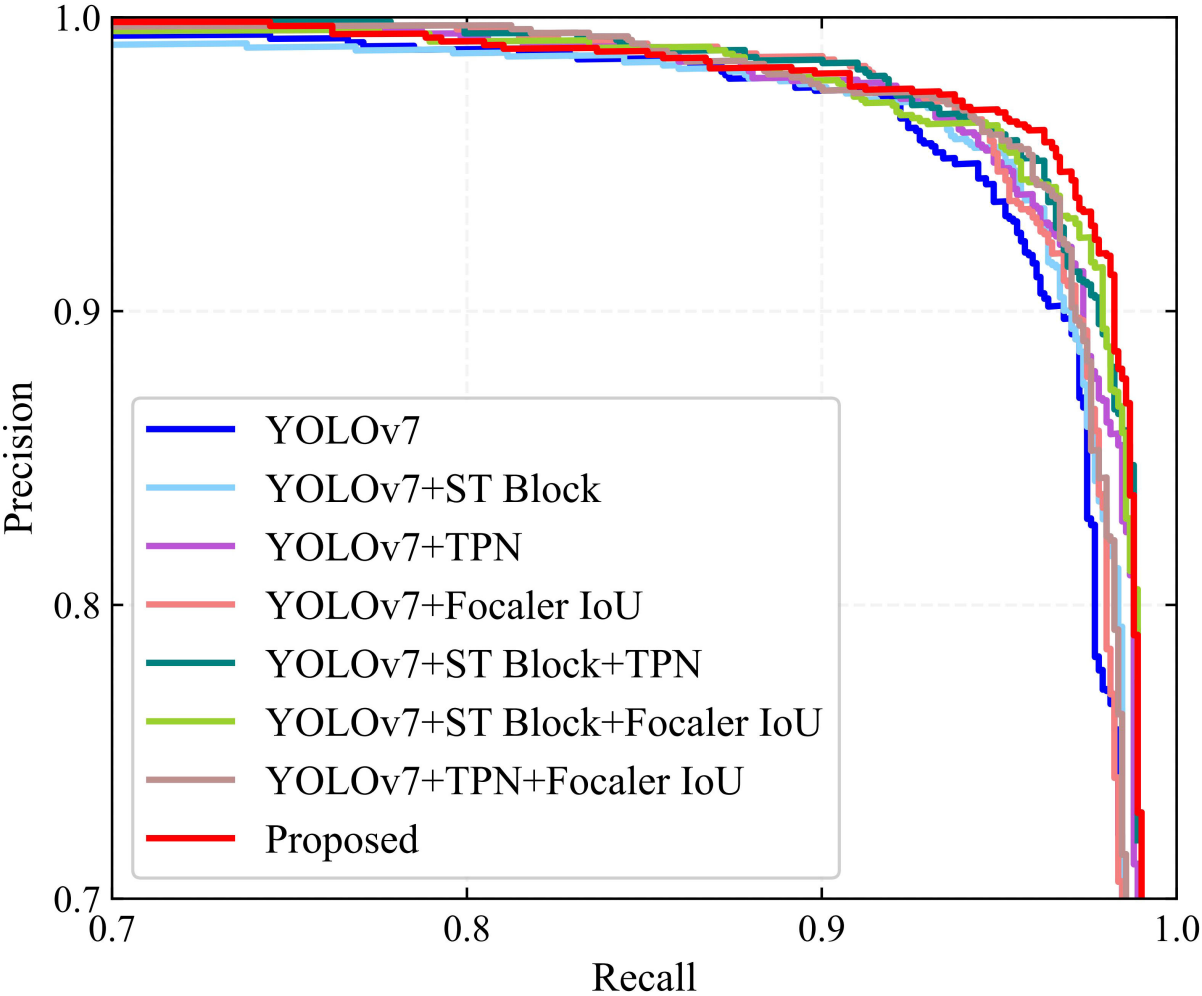
PR curves of the major components of YOLO-SwinTF for ablation study.

This study integrated three components, ST block, TPN, and Focaler-IoU, into the detection model to enhance its performance. An ablation study was conducted to assess the effectiveness of each modification within the proposed model. The results are presented in **Table 3** and **Figure 7**. When the ST block, TPN, and Focaler-IoU are implemented individually, the detection performance improves regarding recall, precision, and AP. Due to the incorporation of the ST block, recall, precision, and AP increased by 0.49%, 0.23%, and 0.19%, respectively, compared to the original YOLOv7 model. This improvement results from the ability to learn global contextual features by establishing long-range dependencies. Including TPN raised the F_1_ score and AP by 0.45% and 0.36%, respectively. Replacing the original IoU with Focaler-IoU led to increases in the F_1_ score and AP of 0.28% and 0.31%, respectively, attributed to the effectiveness of the reconstructed regression loss in handling difficult small targets. The simultaneous use of the ST block and TPN in the model resulted in the F_1_ score and AP of 95.81% and 98.33, increases of 0.51% and 0.35% over using the ST block alone, and 0.42% and 0.18% over using TPN alone. Combining the ST block and Focaler-IoU yielded an increase of 0.21% in both F_1_ score and AP compared to using the ST block alone. When the TPN module was paired with the Focaler-IoU, the F_1_ score and AP reached 95.71% and 98.20%, improvements of 0.32% and 0.05% over using TPN alone and 0.49% and 0.1% over using Focaler-IoU alone. Ultimately, integrating all three modules simultaneously enabled the proposed model to achieve optimal detection performance, with F_1_ score and AP reaching 96.22% and 98.67%, respectively. Therefore, the effectiveness of the three enhancement methods – ST block, TPN, and Focaler-IoU-based regression loss – is verified.

**Table 3:** Ablation study on different components of YOLO-SwinTF.

ST Block	TPN	Focaler-IoU	Recall (%)	Precision (%)	F_1_ (%)	AP (%)
			94.63	95.25	94.94	97.79
✓			95.12	95.48	95.30	97.98
	✓		95.37	95.41	95.39	98.15
		✓	95.05	95.40	95.22	98.10
✓	✓		95.81	95.82	95.81	98.33
✓		✓	95.42	95.60	95.51	98.19
	✓	✓	95.72	95.70	95.71	98.20
✓	✓	✓	96.27	96.17	96.22	98.67

### 4.4 Comparison of different models

**Figure 8:**
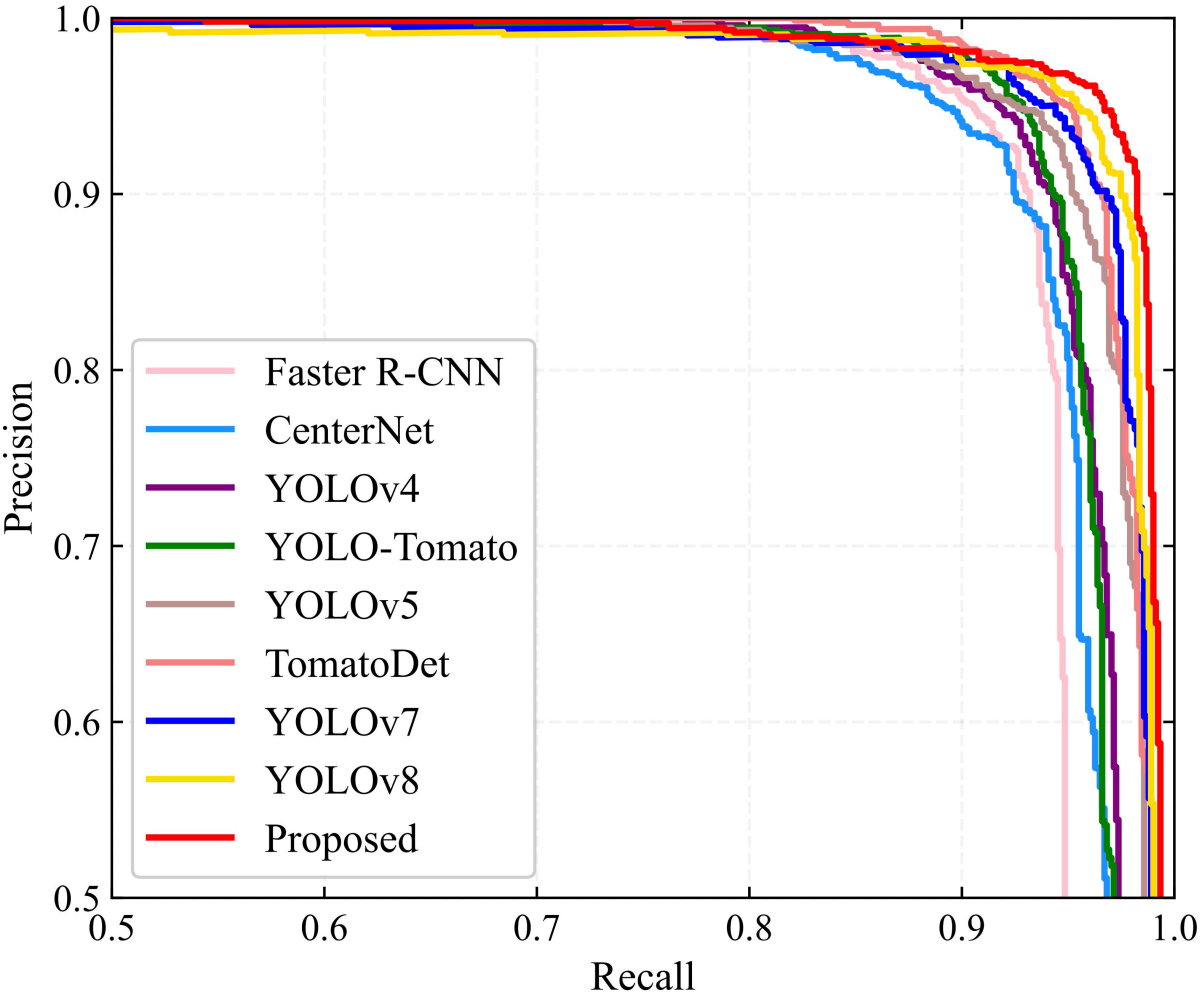
PR curves of different detection algorithms.

A comparative study was conducted alongside leading detection algorithms currently utilized in the field to assess the effectiveness of the newly proposed YOLO-SwinTF model. This study included sophisticated models such as Faster R-CNN (Ren et al., 2015), CenterNet (Zhou et al., 2019), YOLOv4 (Bochkovskiy et al., 2020), YOLO-Tomato (Liu et al., 2020), YOLOv5 (Jocher, 2020), TomatoDet (Liu et al., 2022), YOLOv7 (Wang et al., 2023), YOLOv8 (Jocher et al., 2023), YOLOv9 (Wang et al., 2024b), and YOLOv10 (Wang et al., 2024a). Among these models, Faster R-CNN belongs to the two-stage detection models, whereas the others belong to the single-stage detection models. In addition, CenterNet and TomatoDet are categorized as anchor-free models, while the remaining models rely on anchors for detection. The hyperparameters used for the comparative study, as specified in the original papers (Ren et al., 2015; Zhou et al., 2019; Bochkovskiy et al., 2020; Jocher, 2020; Liu et al., 2020, 2022; Jocher et al., 2023; Wang et al., 2023, 2024a, b), are listed in **Table 4**. **Table 5** displays the detection performance metrics for all detection models, including recall, precision, F_1_ score, AP, and average detection time. Precision-recall (PR) curves are illustrated in **Figure 8**. **Table 5** shows that the proposed model outperforms other methods in all detection metrics, with the exception of detection time. In particular, the YOLO-SwinTF model excels in the F_1_ score and AP, outperforming the second-ranked YOLOv10 by 0.53% and 0.21%, respectively. This improvement primarily benefits from integrating the attention mechanism, TPN architecture, and Focaler-IoU-based loss. However, in terms of detection speed, the YOLO-SwinTF model is 12 ms slower than YOLOv10, primarily due to YOLOv10’s elimination of the post-processing step involving NMS, facilitated by the introduction of dual label assignments. This finding paves the way for our future research. Compared to the baseline model, YOLOv7, the YOLO-SwinTF model shows increases of 1.64% in recall, 0.92% in precision, 1.28% in F_1_ score, and 0.88% in AP, demonstrating the effectiveness of the integrated modules in YOLOv7. The average detection time of the proposed model is 21 ms per image, fulfilling the requirements for real-time tomato detection in complex environments.

**Table 4:** The hyperparameter settings of different algorithms for comparison.

Models	Batch size	Momentum	Weight decay	Initial learning rate	Learning rate decay strategy	Epochs
Faster R-CNN	16	0.9	5 × 10^−4^	10^−3^	Divided by 10 after 90 epochs	120
CenterNet TomatoDet	32	0.9	10^−4^	1.25 × 10^−4^	Divided by 10 after 90 and 120 epochs	140
YOLO- Tomato	32	0.9	5 × 10^−4^	10^−3^	Divided by 10 after 60 and 90 epochs	160
YOLOv4 YOLOv5 YOLOv7 YOLOv8	32	0.937	5 × 10^−4^	10^−3^	Cosine annealing	160
YOLOv9 YOLOv10	32	0.937	5 × 10^−4^	10^−3^	Linear decay	160

**Table 5:** Tomato detection results of different algorithms.

Methods	Recall (%)	Precision (%)	F_1_ (%)	AP (%)	Time (ms)
CenterNet	91.56	92.98	92.26	95.75	32
Faster R-CNN	91.78	92.89	92.33	94.37	231
YOLOv4	92.76	94.11	93.43	93.91	25
YOLO-Tomato	93.09	94.75	93.91	96.40	54
YOLOv5	93.64	94.57	94.10	97.79	22
TomatoDet	94.30	95.77	95.03	98.16	35
YOLOv7	94.63	95.25	94.94	97.79	15
YOLOv8	95.06	95.59	95.32	97.95	12
YOLOv9	95.19	95.71	95.45	98.21	12
YOLOv10	95.55	95.84	95.69	98.46	9
Proposed	96.27	96.17	96.22	98.67	21

### 4.5 Network visualization

**Figure 9:**
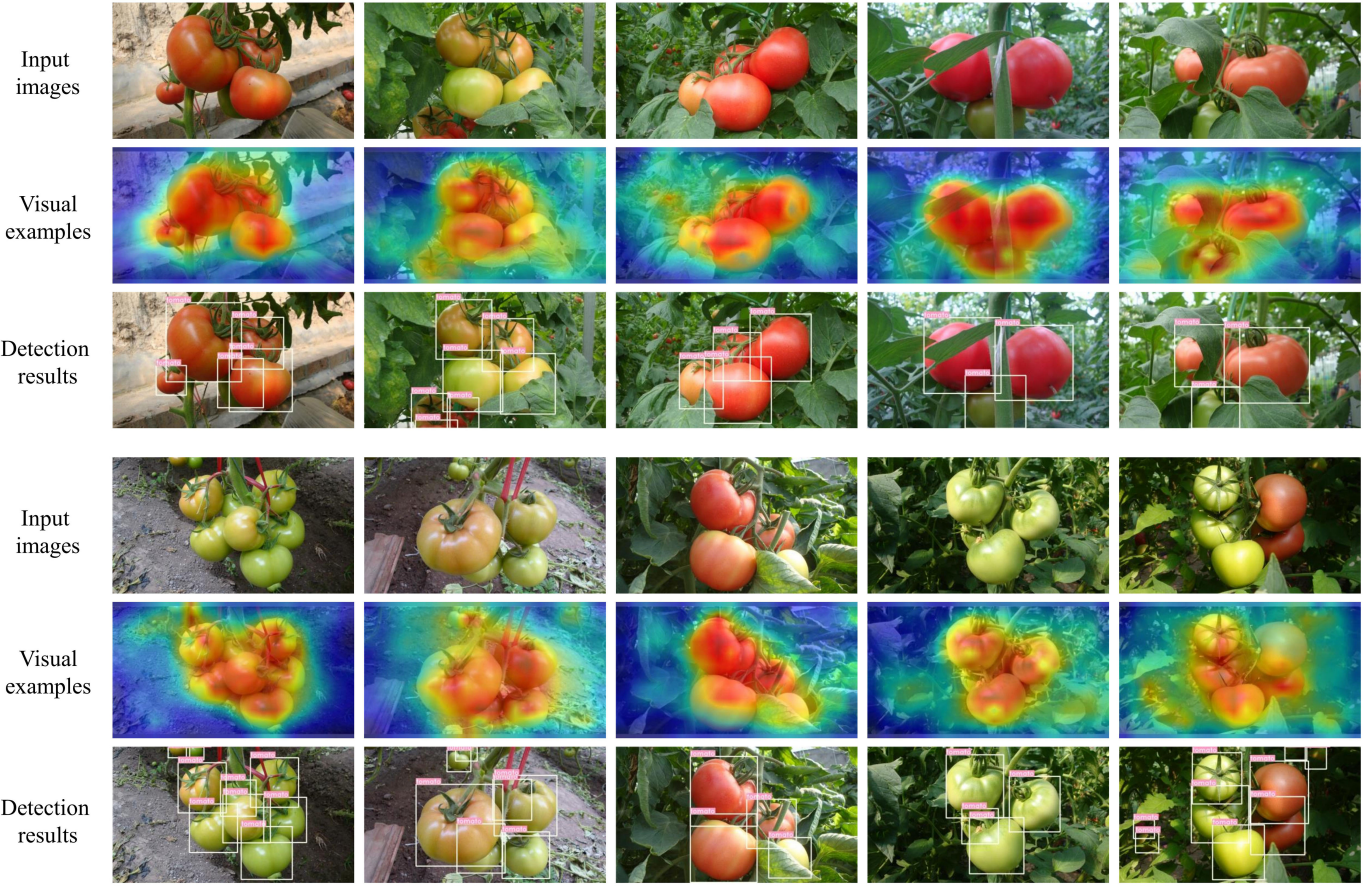
Visual features of images from the tomato dataset.

The Grad-CAM technique (Selvaraju et al., 2017) was employed to visualize the features of raw images to illustrate the superiority of the proposed YOLO-SwinTF intuitively. Specifically, ten images from the tomato dataset were selected, and visual experiments were conducted, as shown in **Figure 9**. The experimental results demonstrate that the image feature extractor, enhanced by the ST block, can capture global information by modeling long-range dependencies and extracting the most significant descriptive content from the raw samples. This capability is primarily attributed to the multi-head self-attention mechanism, which excels in capturing semantic information. In addition, the incorporation of TPN architecture facilitates a better balance between communication-based processing and self-processing, resulting in generating new information for propagation.

### 4.6 Performance of the proposed model under different lighting conditions

**Figure 10:**
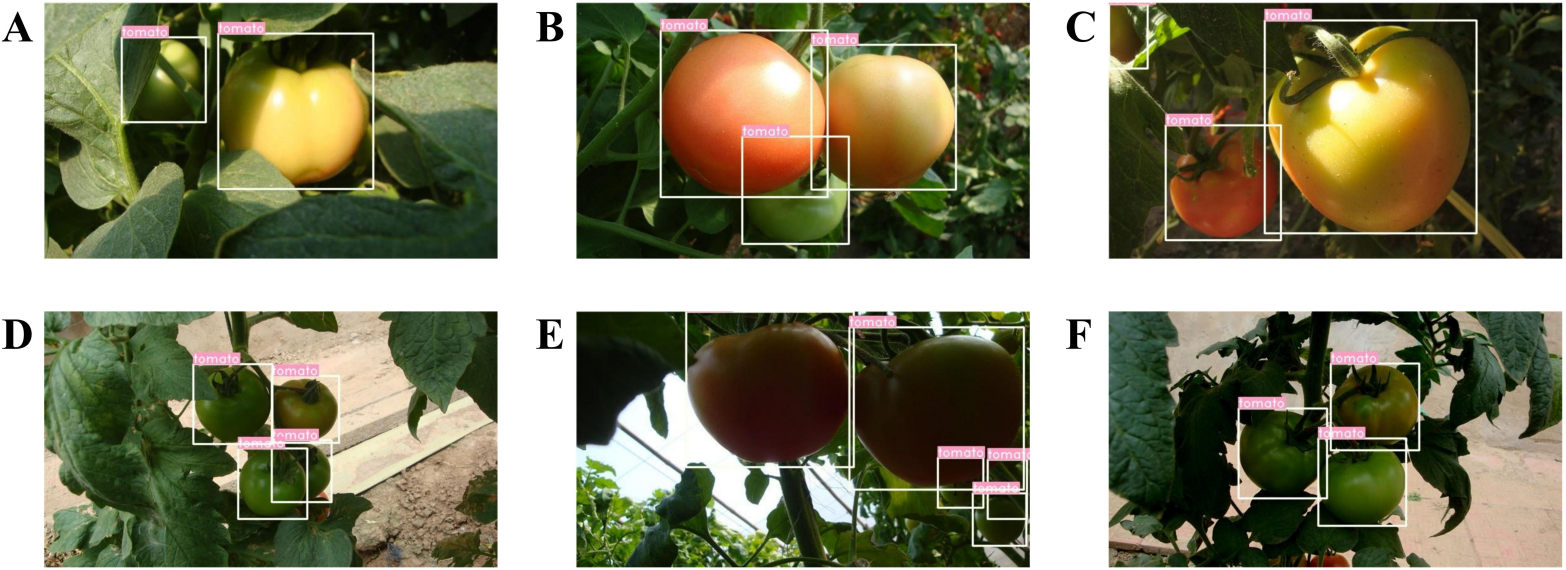
Some examples of the detection results under different lighting conditions: **(A-C)** sunlight conditions, and **(D-F)** shade conditions.

The tomato dataset used in this study was divided into sunlight and shade groups to evaluate the detection performance of the proposed model under different lighting conditions. Of all the test data, 425 tomatoes were in the shade, while the remaining 487 tomatoes were under sunlight. We used the correct identification rate (or recall), false identification rate, and missing rate as the evaluation metrics. The falsely identified tomatoes refer to the detected tomatoes that are actually background, and the term ‘missed tomatoes’ denotes tomatoes that the model did not detect. The detection results are listed in **Table 6**. As shown in **Table 6**, under sunlight conditions, 470 out of 487 tomatoes were correctly detected, with a detection rate of 96.51%. For the shade condition, the detection rate was 96.00%. In addition, under sunlight conditions, some backgrounds were incorrectly identified as tomatoes, with a total of 17 such instances, resulting in an incorrect identification rate of 3.49%. Under the shade condition, the false identification rate was 4.23%. An analysis of the results indicated that these false identifications typically occurred when the tomatoes were similar in shape and color to the background. The above results show that the detection performance of the proposed model is comparable under both sunlight and shade conditions, verifying the robustness of the model to illumination variations. The detection results are shown in **Figure 10**.

**Table 6:** Performance of the proposed model under different lighting conditions.

Illumination	Tomato Count	Correctly Identified		Falsely Identified		Missed	
		Amount	Rate (%)	Amount	Rate (%)	Amount	Rate (%)
Sunlight	487	470	96.51	17	3.49	17	3.49
Shade	425	408	96.00	18	4.23	17	4.00

### 4.7 Performance of the proposed model under different occlusion conditions

**Figure 11:**
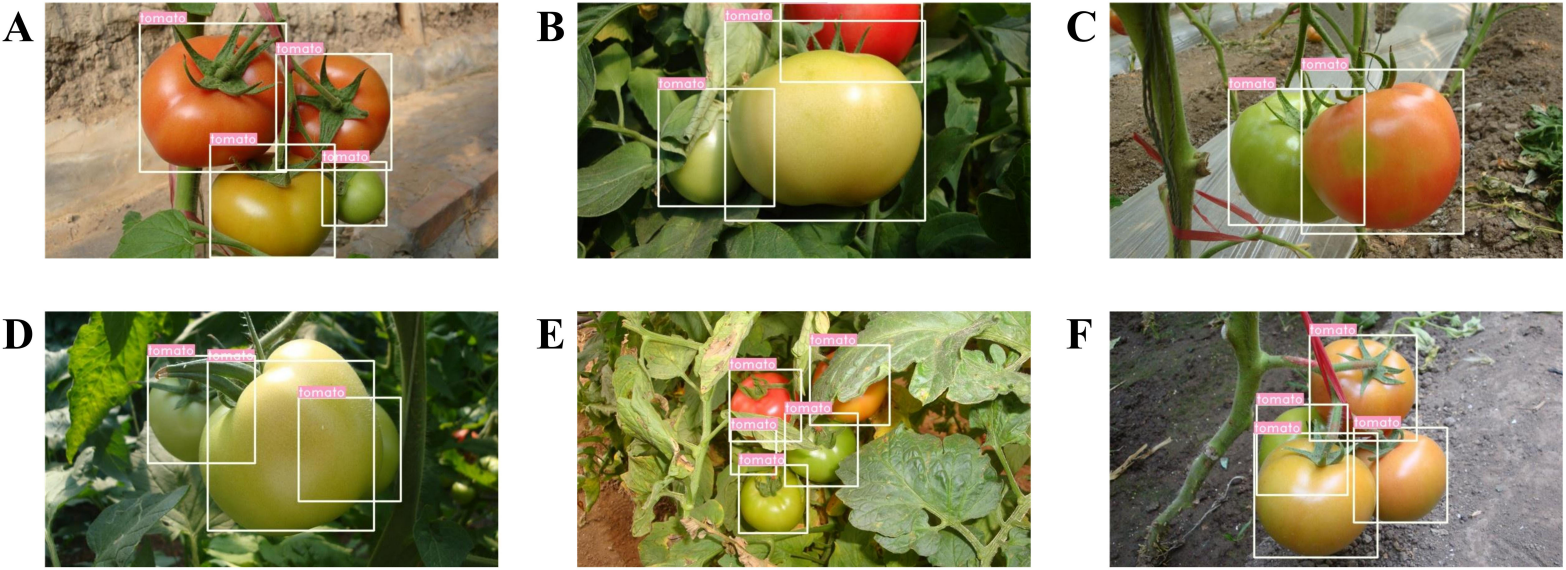
Some examples of detection results under different occlusion conditions: **(A-C)** slight cases and **(D-F)** severe cases.

This study also evaluated the detection performance of the proposed model under different occlusion conditions, which are common in real environments. According to the degree of occlusion of the tomatoes by other objects, tomato data can be categorized into slight and severe occlusion cases. Severe occlusion is defined as the tomatoes being more than 50% occluded by leaves, branches, or other tomatoes, and conversely, they are recognized as slight cases, as defined by Liu et al. (2020). We used the same performance evaluation metrics as in the experiments under different lighting conditions. **Table 7** lists the test results for different occlusion conditions. As shown in **Table 7**, 588 out of 609 tomatoes were correctly identified in the slight occlusion condition, with a detection rate of 96.55%, slightly better than in the severe occlusion condition. The false identification rates in the slight and severe occlusion conditions were 3.45% and 4.61%, respectively, indicating that overlap or occlusion can affect the model’s detection performance. Almost all tomatoes can be detected when the degree of overlap or occlusion is not very severe. The semantic loss of images resulting from overlap or occlusion can be compensated by the model’s attention mechanism and the implicit contextual information mining of hierarchical feature extraction. The model’s performance in detecting overlapping and occluded tomatoes can be further improved by explicitly modeling the contextual environment of tomatoes. **Figure 11** shows some of the detection results.

**Table 7:** Performance of the proposed model under different occlusion conditions.

Occlusion Condition	Tomato Count	Correctly Identified		Falsely Identified		Missed	
		Amount	Rate (%)	Amount	Rate (%)	Amount	Rate (%)
Slight case	609	588	96.55	21	3.45	21	3.45
Severe case	303	290	95.71	14	4.61	13	4.29

## 5 Conclusion

This study proposes a YOLO-SwinTF model designed to enhance the feature expression capabilities of YOLOv7 to achieve accurate tomato detection in complex environments. Initially, the backbone network of the proposed framework incorporates Swin Transformer modules to represent global information by modeling long-range visual dependencies. Subsequently, in the neck network, the TPN architecture replaces the PANet to better balance communication-based processing and self-processing, generating new information for delivery in the feature map. Finally, a novel regression loss based on Focaler-IoU is constructed in bounding box regression to allow the loss function to dynamically adjust its focus between easy and difficult samples, enhancing the model’s detection performance.

Extensive experiments are conducted to verify the performance of the proposed method. The F_1_ score and AP of the proposed YOLO-SwinTF model reached 96.22% and 98.67%, respectively, surpassing other state-of-the-art detectors. Ablation studies are performed to verify the effectiveness of each modification. In addition, the model demonstrates strong robustness in detecting tomatoes under various illumination and occlusion conditions. The experimental results confirm the proposed model is highly suitable for tomato detection in complex environments.

In the future, the ripeness information of tomatoes at different growth stages will be utilized to achieve multi-stage tomato detection. In addition, we plan to implement explicit context modeling for tomatoes to improve the detection performance of overlapping and occluded tomatoes.

## Data Availability

The original contributions presented in the study are included in the article/supplementary material. Further inquiries can be directed to the corresponding author.

## References

[B1] Ashtiani S.-H. M. Javanmardi S. Jahanbanifard M. Martynenko A. Verbeek F. J. (2021). Detection of mulberry ripeness stages using deep learning models. IEEE Access 9, 100380–100394. doi: 10.1109/ACCESS.2021.3096550

[B2] Bargoti S. Underwood J. (2017). “Deep fruit detection in orchards,” in 2017 IEEE international conference on robotics and automation (ICRA), Singapore, 3626–3633 (IEEE).

[B3] Behera S. K. Rath A. K. Sethy P. K. (2020). Fruit recognition using support vector machine based on deep features. Karbala Int. J. Modern Sci. 6, 16. doi: 10.33640/2405-609X.1675

[B4] Bochkovskiy A. Wang C.-Y. Liao H.-Y. M. (2020). Yolov4: Optimal speed and accuracy of object detection. arXiv preprint arXiv:2004.10934. doi: 10.48550/arXiv.2004.10934

[B5] Chaivivatrakul S. Dailey M. N. (2014). Texture-based fruit detection. Precis. Agric. 15, 662–683. doi: 10.1007/s11119-014-9361-x

[B6] Chen J. Kao S.-h. He H. Zhuo W. Wen S. Lee C.-H.. (2023). “Run, don’t walk: Chasing higher flops for faster neural networks,” in Proceedings of the IEEE/CVF Conference on Computer Vision and Pattern Recognition. Vancouver, Canada: IEEE, 12021–12031.

[B7] Ding X. Zhang X. Han J. Ding G. (2022). “Scaling up your kernels to 31x31: Revisiting large kernel design in cnns,” in Proceedings of the IEEE/CVF conference on computer vision and pattern recognition. Lousiana, New Orleans: IEEE, 11963–11975.

[B8] Ding X. Zhang X. Ma N. Han J. Ding G. Sun J. (2021). “Repvgg: Making vgg-style convnets great again,” in Proceedings of the IEEE/CVF conference on computer vision and pattern recognition. Nashville, TN, USA: IEEE, 13733–13742.

[B9] Everingham M. Van Gool L. Williams C. K. Winn J. Zisserman A. (2010). The pascal visual object classes (voc) challenge. Int. J. Comput. Vision 88, 303–338. doi: 10.1007/s11263-009-0275-4

[B10] Fuentes A. Yoon S. Kim S. C. Park D. S. (2017). A robust deep-learning-based detector for real-time tomato plant diseases and pests recognition. Sensors 17, 2022. doi: 10.3390/s17092022 28869539 PMC5620500

[B11] Ganesh P. Volle K. Burks T. Mehta S. (2019). Deep orange: Mask r-cnn based orange detection and segmentation. Ifac-papersonline 52, 70–75. doi: 10.1016/j.ifacol.2019.12.499

[B12] Gao C. Jiang H. Liu X. Li H. Wu Z. Sun X.. (2024). Improved binocular localization of kiwifruit in orchard based on fruit and calyx detection using yolov5x for robotic picking. Comput. Electron. Agric. 217, 108621. doi: 10.1016/j.compag.2024.108621

[B13] Guo J. Yang Y. Lin X. Memon M. S. Liu W. Zhang M.. (2023). Revolutionizing agriculture: Real-time ripe tomato detection with the enhanced tomato-yolov7 system. IEEE Access 11, 133086–133098. doi: 10.1109/ACCESS.2023.3336562

[B14] He K. Gkioxari G. Dollár P. Girshick R. (2017). “Mask r-cnn,” in Proceedings of the IEEE international conference on computer vision. Venice, Italy: IEEE, 2961–2969.

[B15] He K. Zhang X. Ren S. Sun J. (2015). Spatial pyramid pooling in deep convolutional networks for visual recognition. IEEE Trans. Pattern Anal. Mach. Intell. 37, 1904–1916. doi: 10.1109/TPAMI.2015.2389824 26353135

[B16] Hernández G. A. A. Olguin J. C. Vasquez J. I. Uriarte A. V. Torres M. C. V. (2023). Detection of tomato ripening stages using yolov3-tiny. arXiv preprint arXiv:2302.00164. doi: 10.48550/arXiv.2302.00164

[B17] Jana S. Parekh R. (2017). “Shape-based fruit recognition and classification,” in Computational Intelligence, Communications, and Business Analytics: First International Conference, CICBA 2017, Kolkata, India, March 24–25, 2017. 184–196 (Springer), *Revised Selected Papers, Part II*.

[B18] Ji W. Zhao D. Cheng F. Xu B. Zhang Y. Wang J. (2012). Automatic recognition vision system guided for apple harvesting robot. Comput. Electric. Eng. 38, 1186–1195. doi: 10.1016/j.compeleceng.2011.11.005

[B19] Jia W. Xu Y. Lu Y. Yin X. Pan N. Jiang R.. (2023). An accurate green fruits detection method based on optimized yolox-m. Front. Plant Sci. 14, 1187734. doi: 10.3389/fpls.2023.1187734 37223802 PMC10200941

[B20] Jiao Y. Luo R. Li Q. Deng X. Yin X. Ruan C.. (2020). Detection and localization of overlapped fruits application in an apple harvesting robot. Electronics 9, 1023. doi: 10.3390/electronics9061023

[B21] Jocher G. (2020). YOLOv5 by Ultralytics. doi: 10.5281/zenodo.3908559

[B22] Jocher G. Chaurasia A. Qiu J. (2023). Ultralytics YOLO.

[B23] Kelman E. E. Linker R. (2014). Vision-based localisation of mature apples in tree images using convexity. Biosyst. Eng. 118, 174–185. doi: 10.1016/j.biosystemseng.2013.11.007

[B24] Krizhevsky A. Sutskever I. Hinton G. E. (2012). Imagenet classification with deep convolutional neural networks. Adv. Neural Inf. Process. Syst. 25, 1097–1105. doi: 10.1145/3065386

[B25] Kurtulmus F. Lee W. S. Vardar A. (2011). Green citrus detection using ‘eigenfruit’, color and circular gabor texture features under natural outdoor conditions. Comput. Electron. Agric. 78, 140–149. doi: 10.1016/j.compag.2011.07.001

[B26] Lam E. Y. (2005). “Combining gray world and retinex theory for automatic white balance in digital photography,” in Proceedings of the Ninth International Symposium on Consumer Electronics, 2005.(ISCE 2005), Macau, China. 134–139 (IEEE).

[B27] Li C. Zhou A. Yao A. (2022). Omni-dimensional dynamic convolution. arXiv preprint arXiv:2209.07947. doi: 10.48550/arXiv.2209.07947

[B28] Li L. Tang S. Deng L. Zhang Y. Tian Q. (2017). “Image caption with global-local attention,” in Proceedings of the AAAI conference on artificial intelligence. San Francisco, California, USA: AAAI Press, Vol. 31.

[B29] Liu G. Hou Z. Liu H. Liu J. Zhao W. Li K. (2022). Tomatodet: Anchor-free detector for tomato detection. Front. Plant Sci. 13, 942875. doi: 10.3389/fpls.2022.942875 35991435 PMC9389331

[B30] Liu G. Mao S. Kim J. H. (2019). A mature-tomato detection algorithm using machine learning and color analysis. Sensors 19, 2023. doi: 10.3390/s19092023 31052169 PMC6539546

[B31] Liu G. Nouaze J. C. Touko Mbouembe P. L. Kim J. H. (2020). Yolo-tomato: A robust algorithm for tomato detection based on yolov3. Sensors 20, 2145. doi: 10.3390/s20072145 32290173 PMC7180616

[B32] Liu S. Qi L. Qin H. Shi J. Jia J. (2018). “Path aggregation network for instance segmentation,” in Proceedings of the IEEE conference on computer vision and pattern recognition. Salt Lake City, UT, USA: IEEE, 8759–8768.

[B33] Liu W. Anguelov D. Erhan D. Szegedy C. Reed S. Fu C.-Y.. (2016). “Ssd: Single shot multibox detector,” in European conference on computer vision. 21–37 (Amsterdam, The Netherlands: Springer), Proceedings, Part I 14.

[B34] Liu Z. Lin Y. Cao Y. Hu H. Wei Y. Zhang Z.. (2021). Swin transformer: Hierarchical vision transformer using shifted windows. Proc. IEEE/CVF Int. Conf. Comput. vision., 10012–10022. doi: 10.1109/ICCV48922.2021.00986

[B35] Mbouembe P. L. T. Liu G. Sikati J. Kim S. C. Kim J. H. (2023). An efficient tomato-detection method based on improved yolov4-tiny model in complex environment. Front. Plant Sci. 14, 1150958. doi: 10.3389/fpls.2023.1150958 37077640 PMC10106724

[B36] Payne A. Walsh K. Subedi P. Jarvis D. (2014). Estimating mango crop yield using image analysis using fruit at ‘stone hardening’stage and night time imaging. Comput. Electron. Agric. 100, 160–167. doi: 10.1016/j.compag.2013.11.011

[B37] Peng H. Shao Y. Chen K. Deng Y. Xue C. (2018). Research on multi-class fruits recognition based on machine vision and svm. IFAC-PapersOnLine 51, 817–821. doi: 10.1016/j.ifacol.2018.08.094

[B38] Picron C. Tuytelaars T. (2022). Trident pyramid networks for object detection. Proc. BMVC, p. 241.

[B39] Qi Y. He Y. Qi X. Zhang Y. Yang G. (2023). “Dynamic snake convolution based on topological geometric constraints for tubular structure segmentation,” in Proceedings of the IEEE/CVF International Conference on Computer Vision. Paris, France: IEEE, 6070–6079.

[B40] Rakun J. Stajnko D. Zazula D. (2011). Detecting fruits in natural scenes by using spatial-frequency based texture analysis and multiview geometry. Comput. Electron. Agric. 76, 80–88. doi: 10.1016/j.compag.2011.01.007

[B41] Redmon J. Divvala S. Girshick R. Farhadi A. (2016). “You only look once: Unified, real-time object detection,” in Proceedings of the IEEE conference on computer vision and pattern recognition. Las Vegas, NV, USA: IEEE, 779–788.

[B42] Redmon J. Farhadi A. (2017). Yolo9000: better, faster, stronger. Proc. IEEE Conf. Comput. Vision Pattern Recognit., 7263–7271. doi: 10.1109/CVPR.2017.690

[B43] Redmon J. Farhadi A. (2018). Yolov3: An incremental improvement. arXiv preprint arXiv:1804.02767. doi: 10.48550/arXiv.1804.02767

[B44] Ren S. He K. Girshick R. Sun J. (2015). Faster r-cnn: Towards real-time object detection with region proposal networks. Adv. Neural Inf. Process. Syst. 39 (6), 1137–1149. doi: 10.1109/TPAMI.2016.2577031 27295650

[B45] Sa I. Ge Z. Dayoub F. Upcroft B. Perez T. McCool C. (2016). Deepfruits: A fruit detection system using deep neural networks. sensors 16, 1222. doi: 10.3390/s16081222 27527168 PMC5017387

[B46] Samajpati B. J. Degadwala S. D. (2016). “Hybrid approach for apple fruit diseases detection and classification using random forest classifier,” in 2016 International conference on communication and signal processing (ICCSP) (IEEE). Melmaruvathur, India: IEEE, 1015–1019.

[B47] Selvaraju R. R. Cogswell M. Das A. Vedantam R. Parikh D. Batra D. (2017). “Grad-cam: Visual explanations from deep networks via gradient-based localization,” in Proceedings of the IEEE international conference on computer vision. Venice, Italy: IEEE, 618–626.

[B48] Tan M. Le Q. (2019). “Efficientnet: Rethinking model scaling for convolutional neural networks,” in International conference on machine learning (PMLR). Long Beach, California, USA: PMLR Press, 6105–6114.

[B49] Wang A. Chen H. Liu L. Chen K. Lin Z. Han J.. (2024a). Yolov10: Real-time end-to-end object detection. arXiv preprint arXiv:2405.14458. doi: 10.48550/arXiv.2405.14458

[B50] Wang C.-Y. Bochkovskiy A. Liao H.-Y. M. (2023). “Yolov7: Trainable bag-of-freebies sets new state-of-the-art for real-time object detectors,” in Proceedings of the IEEE/CVF conference on computer vision and pattern recognition. Vancouver, Canada: IEEE, 7464–7475.

[B51] Wang C.-Y. Liao H.-Y. M. Wu Y.-H. Chen P.-Y. Hsieh J.-W. Yeh I.-H. (2020). “Cspnet: A new backbone that can enhance learning capability of cnn,” in Proceedings of the IEEE/CVF conference on computer vision and pattern recognition workshops. Seattle, WA, USA: IEEE, 390–391.

[B52] Wang C.-Y. Liao H.-Y. M. Yeh I.-H. (2022). Designing network design strategies through gradient path analysis. arXiv preprint arXiv:2211.04800. doi: 10.48550/arXiv.2211.04800

[B53] Wang C.-Y. Yeh I.-H. Liao H.-Y. M. (2024b). Yolov9: Learning what you want to learn using programmable gradient information. arXiv preprint arXiv:2402.13616. doi: 10.48550/arXiv.2402.13616

[B54] Wang W. Shi Y. Liu W. Che Z. (2024c). An unstructured orchard grape detection method utilizing yolov5s. Agriculture 14, 262. doi: 10.3390/agriculture14020262

[B55] Wei X. Jia K. Lan J. Li Y. Zeng Y. Wang C. (2014). Automatic method of fruit object extraction under complex agricultural background for vision system of fruit picking robot. Optik 125, 5684–5689. doi: 10.1016/j.ijleo.2014.07.001

[B56] Yu L. Xiong J. Fang X. Yang Z. Chen Y. Lin X.. (2021). A litchi fruit recognition method in a natural environment using rgb-d images. Biosyst. Eng. 204, 50–63. doi: 10.1016/j.biosystemseng.2021.01.015

[B57] Zeng T. Li S. Song Q. Zhong F. Wei X. (2023). Lightweight tomato real-time detection method based on improved yolo and mobile deployment. Comput. Electron. Agric. 205, 107625. doi: 10.1016/j.compag.2023.107625

[B58] Zhang H. Zhang S. (2024). Focaler-iou: More focused intersection over union loss. arXiv preprint arXiv:2401.10525. doi: 10.48550/arXiv.2401.10525

[B59] Zhao Y. Gong L. Zhou B. Huang Y. Liu C. (2016). Detecting tomatoes in greenhouse scenes by combining adaboost classifier and colour analysis. Biosyst. Eng. 148, 127–137. doi: 10.1016/j.biosystemseng.2016.05.001

[B60] Zheng Z. Wang P. Liu W. Li J. Ye R. Ren D. (2020). “Distance-iou loss: Faster and better learning for bounding box regression,” in Proceedings of the AAAI conference on artificial intelligence. New York, USA: AAAI Press, Vol. 34. 12993–13000.

[B61] Zhou X. Wang D. Krähenbühl P. (2019). Objects as points. arXiv preprint arXiv:1904.07850. doi: 10.48550/arXiv.1904.07850

[B62] Zhu H. Yang L. Fei J. Zhao L. Han Z. (2021). Recognition of carrot appearance quality based on deep feature and support vector machine. Comput. Electron. Agric. 186, 106185. doi: 10.1016/j.compag.2021.106185

